# Practical Notes on Diagnosis and Treatment

**Published:** 1907-06-15

**Authors:** 


### Practical Notes on Diagnosis and Treatment.

**Nocturnal Asthma.**

Morphine $\frac{1}{10}$ grain with atropine sulphate $\frac{1}{200}$ grain is a most efficient agent in patients suffering from nightly attacks of cardiac asthma.

**Abscess of the Brain.**

Sir Samuel Wilks pointed out many years ago that in sub-acute abscess of the brain the temperature, so far from being raised, is generally subnormal.

**Cirrhosis of the Liver.**

A large liver with cirrhosis is sometimes due to infiltration of the cells of the liver with fat. A hard and rounded edge of the liver is the most important physical sign of cirrhosis, especially in the early stage before the development of ascites.

**Opium Poisoning.**

In addition to the usual remedial measures, permanganate of potassium may be given; dissolve 8 grains in an ounce of water and give a teaspoonful every ten minutes, increasing the interval with the improvement of the patient. As much as 16 grains given in this way has been followed by recovery.

**Mercury by Subcutaneous Injection.**

A very good way when the rapid action of mercury is required (as in syphilis of the central nervous system) is to dissolve $\frac{1}{8}$ grain of the perchloride in 5 minims of water and to inject this deeply into the gluteal region once a day. This usually suffices to keep a patient just on the brink of salivation.—*Dr. Hale White.*

